# Wear mechanisms of thin dental composites

**DOI:** 10.3389/fbioe.2025.1545026

**Published:** 2025-03-27

**Authors:** Magdalena A. Osiewicz, Arie Werner, Franciscus J. M. Roeters, Cornelis J. Kleverlaan

**Affiliations:** ^1^ Department of Integrated Dentistry, Jagiellonian University, Krakow, Poland; ^2^ Department of Dental Materials Science, Academic Centre for Dentistry Amsterdam (ACTA), University of Amsterdam and Vrije Universiteit Amsterdam, Amsterdam, Netherlands; ^3^ Department of Comprehensive Dentistry, Academic Centre for Dentistry Amsterdam (ACTA), University of Amsterdam and Vrije Universiteit Amsterdam, Amsterdam, Netherlands

**Keywords:** wear, fatigue wear, scanning electron microscopy, composite resin, bruxism

## Abstract

**Objective:**

In patients with severe wear, the performance of restorative materials is challenging, especially in load-bearing thin restorations. In this study, we aimed to investigate the wear rate in thin-layered restoration (1.5 mm) compared to more bulky restorations (3 mm), where less deformation and stress within the material are expected.

**Methods:**

The wear rates of four resin-based composites were measured using one layer of 3-mm thickness compared to a thin-layered specimen of 1.5-mm composite, which was supported by a flexible layer of 1.5-mm silicone impression material. Two- and three-body wear were measured using the ACTA wear device. Scanning electron microscopy analysis was performed to detect the surface alterations. One- and two-way ANOVA and Tukey’s *post hoc* test were used to analyze differences in wear values.

**Results:**

The two-body wear of the 1.5-mm specimens was significantly higher (p < 0.001) than that of the 3-mm specimens. The increase in the wear rate between the 1.5-mm and 3-mm specimens can be attributed to fatigue wear. The three-body wear of the 1.5-mm Heliomolar (HMR) specimens was significantly higher than that of the 3-mm HMR specimens. However, for the three-body wear, there was no significant difference between the 1.5-mm and 3-mm specimens of Clearfil AP-X and Clearfil Majesty ES-2.

**Significance:**

The results of this study show for the first time that fatigue wear plays a role in the wear mechanism of thin (1.5 mm) dental resin-based composites. Therefore, the deformation of restorations under loading should be minimized by avoiding thin restorations and flexible conditions and using resin-based composites with high E-moduli.

## Introduction

Dental wear can occur due to various factors such as chewing, tooth brushing, and consumption of acidic foods and drinks. When restoration is needed, wear rarely poses an issue in the majority of cases. However, in patients with parafunctional habits such as bruxism, even the performance of all restorative materials becomes challenging. Sleep and awake bruxism are masticatory muscle activities that result in repetitive or sustained tooth contact (Lobbezoo et al., 2018). The prevalence of bruxism varies in different reports, ranging from 5% to 91% (Lobbezoo et al., 2012). However, the prevalence of sleep bruxism is usually considered to be approximately 8%–13% of the general population (Manfredini et al., 2013). Sleep bruxism is more common in children (14%–18%) and least common in the elderly (approximately 3%). Awake bruxism is more prevalent than sleep bruxism, with rates ranging from 15% to 32% (Archer et al., 2023; Oliveira et al., 2023).

**FIGURE 1:**
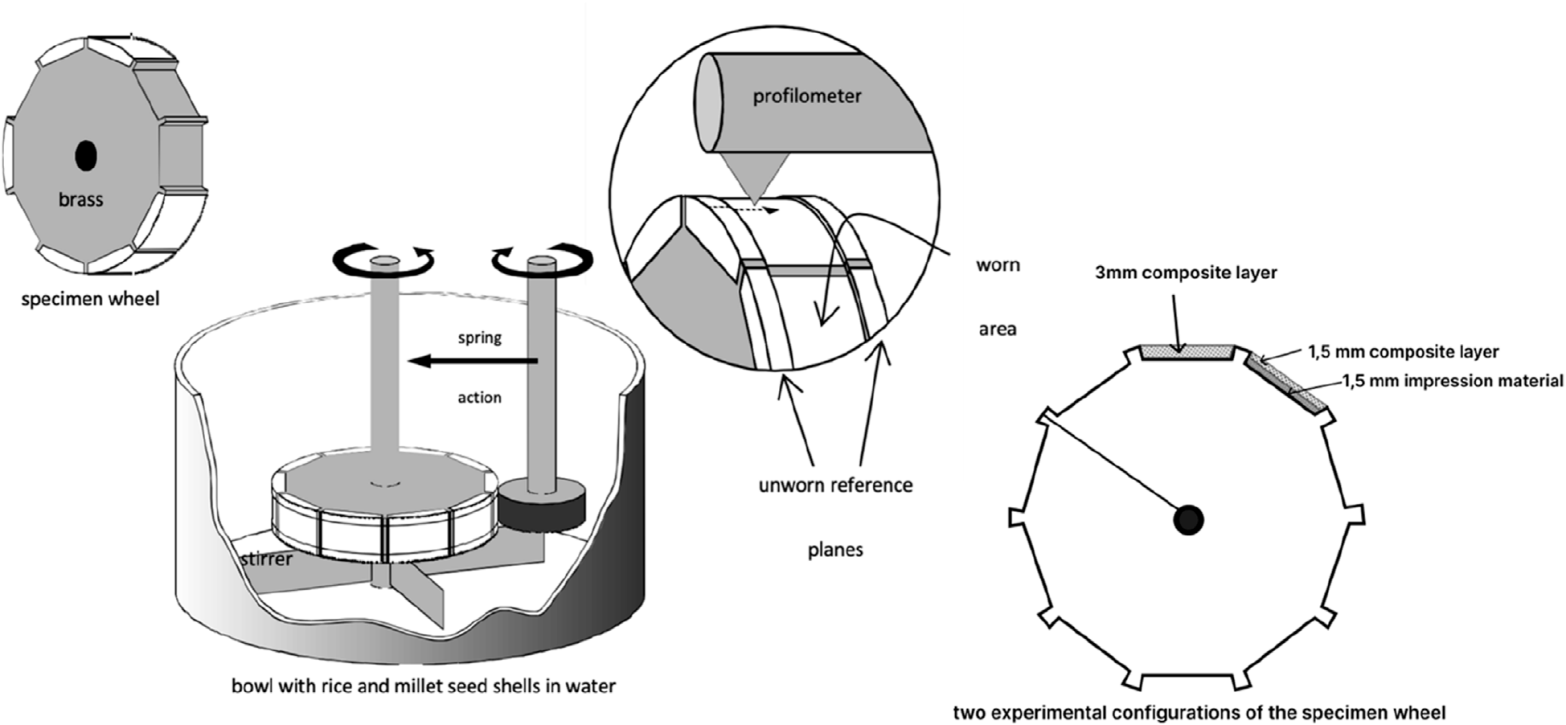
Schematic representation of the various steps in the wear experiment.

As bruxism is common, teeth and restorations frequently show high wear rates in these patients. The wear of composite restorations can be described from an engineering point of view by four main mechanisms (Mair, 1992). *Abrasive* *wear* ^1^ can be subdivided into two-body wear (sandpaper) and three-body wear (polishing paste); *adhesive wear* ^2^ is related to friction between two surfaces and involves local cold welding of the material; *fatigue wear* ^3^ is a result of the formation and propagation of subsurface microcracks as surfaces move under dynamic load (see Figure 1); and *erosive wear* ^7^ is commonly the result of particles from fluids under pressure (sandblasting). However, in dentistry, the following terminology is commonly used: *attrition* refers to the wear of teeth at sites from direct contact between teeth or restorations (flattening of cup tips and slopes). *Erosion* results from surface loss due to etching by acidic foods or fluids. Furthermore, *abrasion* can occur due to an abrasive diet or parafunctional habits, such as excessive biting of teeth/nails or other objects or other oral habits. In patients with parafunctions such as bruxism, two mechanisms play a role in the wear of resin composites. This is abrasion due to the direct contact in two- and three-body wear. However, fatigue wear may also be involved in the wear of dental composites, explaining the high wear rates in bruxism patients for some composite materials. Fatigue wear has been previously postulated by different authors (Gee and Pallav, 1994; Heintze et al., 2019; Mair, 1992), but clear proof that fatigue wear plays a role *in vivo* or *in vitro* in the wear of resin composites has not been clearly demonstrated. Clinical studies of patients with extreme wear by Bartlett and Sundaram (2006) showed that microfilled composites were not considered to be strong enough to restore occlusion. However, a retrospective study using the hybrid resin composites Clearfil AP-X and Clearfil Photo Bright^11^ showed acceptable results. This raised the question of whether the type of composite plays a role in the wear and ultimately the survival of restorations.

In fatigue wear, areas with direct contact and located slightly below the surface are those where stresses and deformations are confined (Gee and Pallav, 1994; Juvinall and Marshek, 1991). Subsurface cracks can eventually lead to surface damage and high wear rates. The deformation of a restoration is not only related to the stress but also to the stiffness of the material and the flexibility of the supporting material. In the previous study (Osiewicz et al., 2022), we investigated layered systems and expected a higher wear rate when the supporting material was more flexible, and a lower wear rate was observed when the supporting material was relatively stiff because of reduced deformation of the top layer. In the latter study, the supporting material was a bulk-fill resin composite that was too stiff to show a fatigue wear effect (Osiewicz et al., 2022). However, if stresses and deformations are high, fatigue wear can play a role in the overall wear of resin composites (Gee and Pallav, 1994; Mair, 1992).

This *in vitro* study aimed to provide insight into the wear mechanism of thin-layered resin composite restorations (1.5 mm) compared to the intrinsic wear rate of bulkier resin composite restorations (3.0 mm).

## Materials and methods

The materials used in the study were Clearfil AP-X (AP-X), Clearfil Photo Bright (CPB), Heliomolar (HMR), Filtek Universal Restorative (FURE), and Clearfil Majesty Esthetic 2 (CME2). The composition, type of composite, manufacturers, batch numbers, expiration date, color, and E-moduli are shown in Table 1.

**TABLE 1:** Properties of the materials used in the experiment according to the manufacturer’s data.

Code	Material (type)	Composition	Batch/exp/color	E-modulus (in GPa)
AP-X	Clearfil AP-X^a^ (Microhybrid)	Bis-GMA, TEGDMA, silanated barium glass filler, silanated silica filler, silanated colloidal silica, and dl-camphorquinone	4P0712 2022-03/A2	15.3^a^/16.7^b^
CPB	Clearfil Photo Bright^a^ (Hybrid)	Bis-GMA, silanated colloidal silica, pre-polymerized organic filler containing colloidal silica, dimethacrylates, photoinitiator, and others	42,001 2021-09/A1	11.5^b^
FURE	Filtek Universal Restorative^b^ (Nanohybrid)>	AUDMA, AFM, silica filler, zirconia filler, and ytterbium trifluoride filler	NA27754 2022-01/A2	N/A
HMR	Heliomolar^c^ (Microfill)	Bis-GMA, urethane dimethacrylate, highly dispersed silicone dioxide, pre-polymer, and ytterbium trifluoride	X48,081 2022-11/A1	3.1^c^
CME2	Clearfil Majesty ES-2^a^ (Nanohybrid)	Bis-GMA, dimethacrylate, CQ, silanated barium glass filler, and pre-polymerized organic filler	BJ0033 2023-08/A2	10.0^b^

^a^ Kuraray Dental, Tokyo, Japan.

^b^ 3M ESPE, Seefeld, Germany.

^c^ Ivoclar Vivadent AG, Schaan, Liechtenstein (Boaro et al., 2010; Kuraray, 2023; Thomsen and Peutzfeldt, 2007).

### Two- and three-body wear

Two-- and three-body wear were evaluated using a wear machine developed by the Academic Centre for Dentistry Amsterdam (Gee and Pallav, 1994). The wear machine was equipped with two wheels of different diameters, ∅48 and ∅19 mm, which rotated in the same direction with an approximate 15% difference in the circumferential speed while remaining in close contact with each other (Figure 1). Two-body wear (in μm/200,000 revolutions) was determined when the specimen wheel was in full contact with the antagonist wheel, whereas three-body wear (in μm/200,000 revolutions) was calculated using an abrasive medium—a slurry of rice and millet seeds—as the third body between the two wheels.^4^ Each specimen wheel accommodated 10 specimens, and the experiments were carried out in duplicate. The specimens were placed on the circumference of one wheel, while the other wheel served as an antagonist made of stainless steel with an extra-hardened outer surface. This setup is considered the gold standard according to the ACTA wear protocol ^4^ and is described in ISO/TS14569–2:2001.

All restorative materials were handled and cured according to the manufacturer’s instructions; for example, light-cured (1,400 mW/cm^-2^) for at least 20 s in layers of 2 mm. The specimen wheel accommodated the following materials (in duplicate), and two experimental configurations were evaluated: (i) the compartment of the specimen wheel was filled with a 3.0-mm composite layer, and (ii) the compartment of the specimen wheel was filled with a 1.5-mm layer of impression material (Identium Light, Kettenbach) at the bottom and covered with a 1.5-mm composite layer. The specimen wheels were stored in water at room temperature throughout the experiment. The specimen wheel and the antagonist wheels were kept in water for 2 months prior to the experiment. The diameter of the antagonist wheels was measured at the start and the end of the two-body wear test. The wheels were pressed against each other with a spring force of 15 N. A test run consisted of 200,000 cycles rotating at a speed of 1 Hz, which corresponds to 1 year in real conditions. After the experiment, 10 tracings (n = 10) were taken at fixed positions on the worn surfaces of the specimens (PRK profilometer No. 20702, Perthen GmbH) to determine the loss of material, and the standard deviations were calculated from these profiles.

### Roughness measurements

The surface profile roughness (RA) was measured using a profilometer (SJ-400, Mitutoyo Corporation, Japan) according to the ISO 4287:1997 parameters with Ra—the arithmetic mean of the absolute values of peaks and valleys—measured over a length of 0.8 mm. The worn surfaces were observed via scanning electron microscopy (SEM) at ×1,000 (EVO^®^ LS 15, Analytical environmental SEM, Zeiss). The secondary electron detector (BSED) was used. SEM specimens were indirectly prepared from epoxy resin (Araldite, Ciba-Geigy), which was poured into a polyvinylsiloxane impression and subsequently gold-sputtered to ensure electron conductivity.

### Statistical analysis

Two-way ANOVA was used to test for significant differences in the wear rate, materials, and experimental configuration. One-way ANOVA and Tukey’s *post hoc* test (p < 0.05) were used to evaluate significant differences in the wear rate within materials. SPSS 26 software (SPSS, Chicago, IL, United States) was used for statistical analysis.

## Results

The wear rates (in μm/200,000 revolutions) are summarized in Table 2. The two-way ANOVA of the two-body wear showed significant differences for the experimental setup (1.5 mm vs. 3.0 mm; F = 247.8 and p < 0.001) and resin composites (F = 27.6; p < 0.001), and their interaction was also significantly different (F = 13.9; p < 0.001). The three-body wear was significantly different for the experimental setup (1.5 mm vs. 3.0 mm; F = 35.3 and p < 0.001) and resin composites (F = 1797.7; p < 0.001), and their interaction was also significantly different (F = 161.7; p < 0.001). The two-body wear of the 1.5-mm specimens was always significantly higher than that of the 3.0-mm specimens (p < 0.05). The highest increase in the two-body wear rate was observed for HMR when comparing the 1.5-mm and 3.0-mm specimens (1.9–7.2 μm/200,000 revolutions). The three-body wear of the 1.5-mm specimens of HMR was also significantly higher than that of the 3.0-mm HMR specimens. For the three-body wear, there were no significant differences for AP-X and CME2 (p > 0.05) between the 1.5-mm and 3.0-mm specimens.

**TABLE 2:** Wear rate (in μm/200,000 rev) and standard deviation in parentheses for five resin composites.

2-body wear	AP-X	CPB	HMR	FURE	CME2
3.0 mm	0.9 (1.4)^A^	1.0 (1.5)^A^	1.9 (1.5)^B^	1.4 (1.2)^AB^	1.3 (1.4)^AB^
1.5 mm	2.8 (3.4)^C^	3.9 (1.9)^C^	7.2 (4.2)	2.8 (1.6)^C^	4.1 (2.2)^C^
3-body wear	AP-X	CPB	HMR	FURE	CME2
3.0 mm	33.2 (2.8)^a^	27.1 (1.2)	51.0 (3.1)	32.0 (2.4)	54.0 (3.6)^b^
1.5 mm	33.6 (2.0)^aA^	29.0 (2.0)	56.0 (5.4)	33.7 (2.8)^A^	52.6 (4.5)^b^

The same lowercase letters indicate no significant differences (p > 0.05) between 3.0 mm and 1.5 mm.

The same uppercase letters indicate no significant differences (p > 0.05) between materials.

**FIGURE 2:**
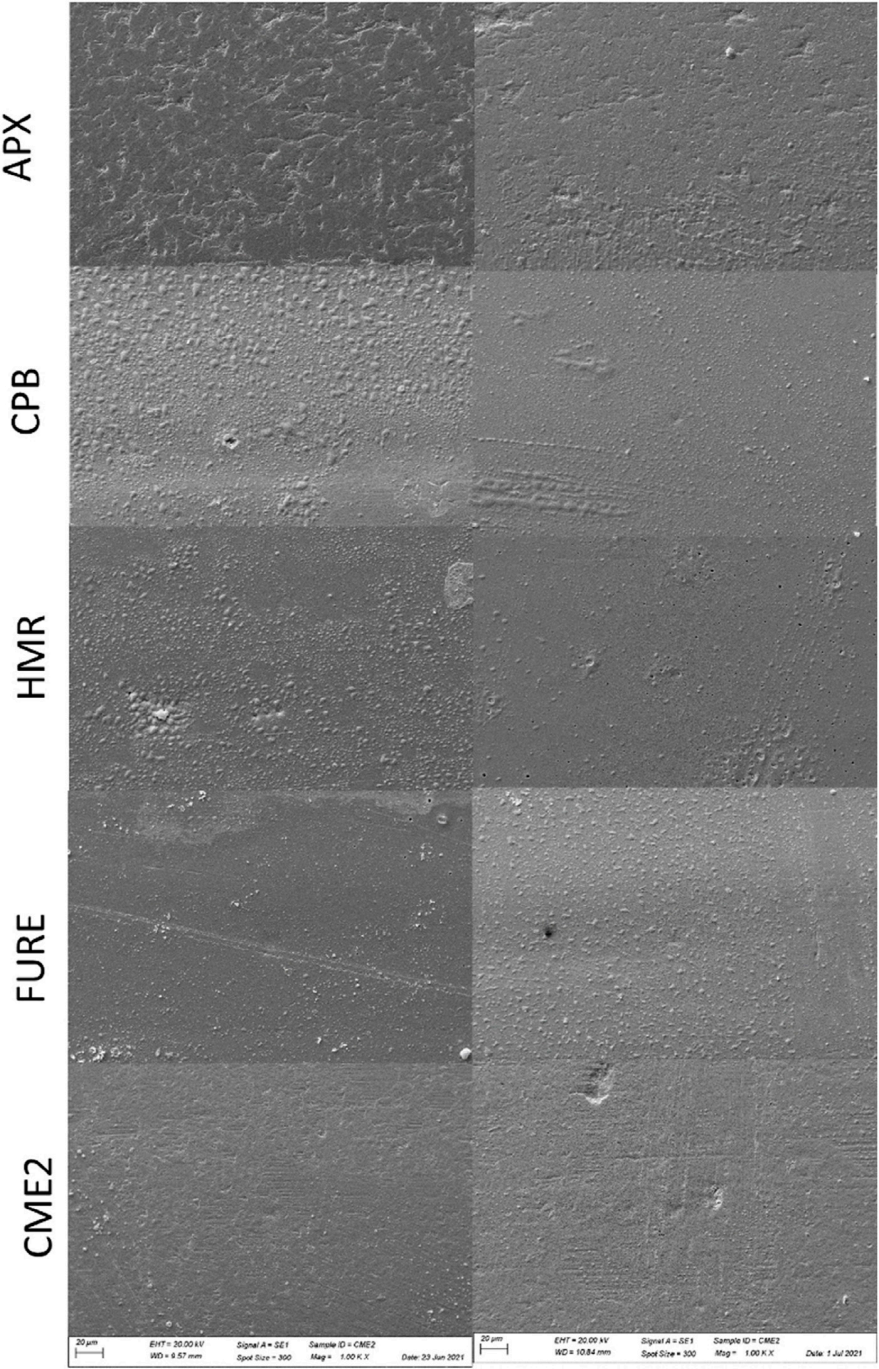
Representative SEM images (1,000×) of the specimens of AP-X, CPB, HMR, FURE, and CME2 after the two-body wear experiment with an antagonist wheel of stainless steel. On the left side, the specimens with a thickness of 1.5 mm; on the right side, the specimens with a thickness of 3.0 mm.

**FIGURE 3:**
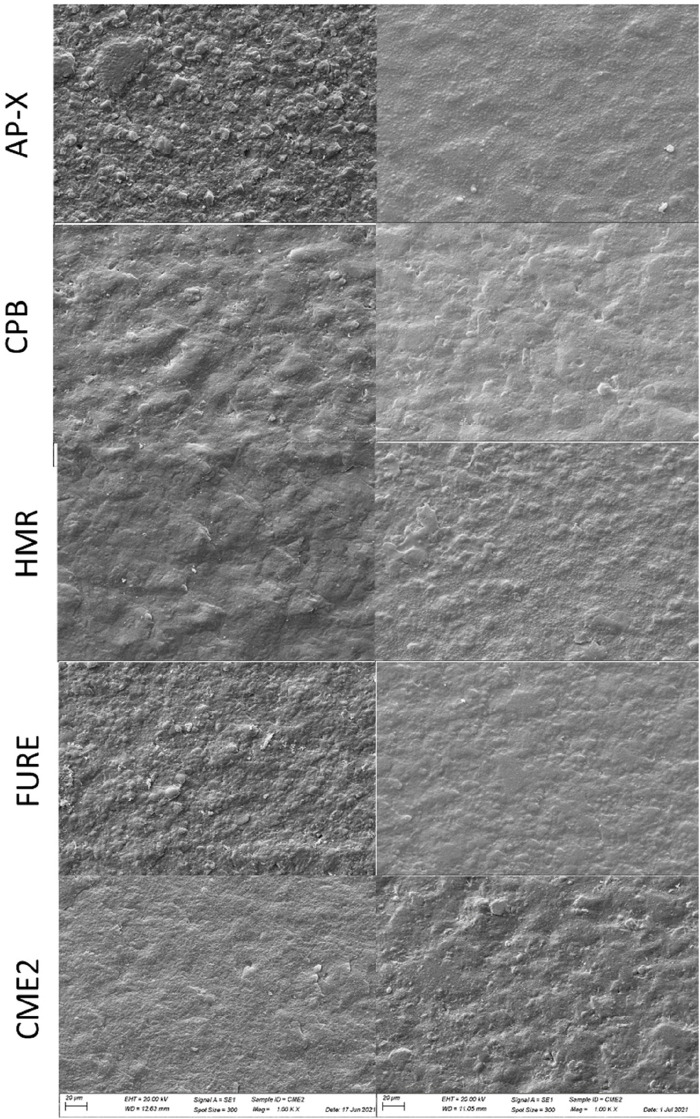
Representative SEM images (1,000×) of the specimens of AP-X, CPB, HMR, FURE, and CME2 after the three-body wear experiment with an antagonist wheel of stainless steel. On the left side, the specimens with a thickness of 1.5 mm; on the right side, the specimens with a thickness of 3.0 mm.

Figures 2, 3 show representative SEM images of the two- and three-body wear (1.5 mm and 3.0 mm) specimens (AP-X). The same pattern was observed for all composites investigated. The surfaces of the specimens from the two- and three-body wear are distinctly different in roughness and SEM. The normal two-body wear generally results in a smooth surface, whereas the normal three-body wear shows a rougher surface with visible particles protruding from the outer surface. The roughness values for the two-body wear were significantly lower than those for the three-body wear in all cases (see Table 3). Comparing the roughness between the 1.5-mm and 3.0-mm setups did not reveal any significant differences. The SEM pictures of the two-body wear between the 1.5-mm and 3.0-mm setup were also comparable. However, the SEM pictures of the 1.5-mm and 3.0-mm setup for the three-body wear revealed a different appearance. For the 1.5-mm setup, the particles appear to be much smoother than those of the 3.0-mm setup; however, this difference was not observed with the Ra values from the profilometer.

**TABLE 3:** Profile roughness (Ra in μm) and standard deviation in parentheses for five resin composites.

2-body wear	AP-X	CPB	HMR	FURE	CME2
3.0 mm	0.21 (0.03)^aA^	0.24 (0.16)^cA^	0.20 (0.01)^dA^	0.21 (0.13)^eA^	0.22 (0.06)^fA^
1.5 mm	0.25 (0.07)^aCD^	0.22 (0.07)^cBC^	0.32 (0.03)^dD^	0.26 (0.09)^eCD^	0.15 (0.01)^fB^
3-body wear	AP-X	CPB	HMR	FURE	CME2
3.0 mm	0.87 (0.08)^b^	1.00 (0.08)^E^	1.04 (0.15)^E^	0.65 (0.03)	0.45 (0.15)
1.5 mm	0.91 (0.06)^bF^	0.80 (0.23)^F^	0.86 (0.15)^F^	0.87 (0.14)^F^	0.85 (0.24)^F^

The same lowercase letters indicate no significant differences (p > 0.05) between 1.5 mm and 3.0 mm.

The same uppercase letters indicate no significant differences (p > 0.05) between materials.

## Discussion

This study showed that fatigue wear plays a role in the two-body wear of the investigated composites. Furthermore, the type of composite had a significant influence on the wear rate of the investigated resin composites. We expected a higher wear rate when the supporting material is more flexible and a lower wear rate when the supporting material is relatively stiff. This is because of the reduced deformation of the top layer and the reduced fatigue wear. The expectations were confirmed under these experimental conditions. It has been proven that fatigue wear plays a role in dental resin-based composites. A thin layer of restoration subjected to heavy loads and materials with relatively low elastic moduli will demonstrate a relatively high wear rate. This experiment demonstrated that the highest increase in the wear rate occurred in the material with the lowest elastic modulus (HRM) and that support from a flexible subsurface further increased the wear rate.

Based on the wear results, AP-X, CPB, and FURE are the preferred materials as they have the lowest three- and two-body wear rates in a thin layer of 1.5 mm. AP-X and FURE are the preferred materials to restore posterior teeth, and CPB can be used as a restorative for anterior teeth. The second best option is CME2, followed by HMR, as HRM showed a significantly higher wear rate for the three- and two-body wear rates in the 1.5 mm layer than the other investigated resin-based composites.

For the majority of resin composites, the clinical wear is more influenced by patient-related factors than by the material itself. Therefore, the majority of the wear studies of the materials are investigated under standardized conditions with an emphasis on the differences between the materials. ^8^ In this study, we showed that the conditions of the experiment and the stiffness of the resin-based composites affected the observed wear, especially in the two-body wear. According to the observed results, restorations in patients with parafunctional habits, such as clenching and grinding, are expected to exhibit a relatively high wear rate, especially when the restorations are less than 1.5 mm thick. Fatigue wear may also explain why restorations in some patients show extremely high wear rates.

The results obtained in this *in vitro* study are partially confirmed by clinical studies of patients treated with resin composites for loss of vertical dimension due to extreme wear, as reported by Bartlett and Sundaram (2006). The agglomerated microfilled composite used in that study was not strong enough to restore occlusion, as after 3 years, 50% of the restorations were lost (28%) or fractured (22%). However, a retrospective study of patients with severe tooth wear using the hybrid resin composites AP-X and CPB ^11^ showed different results. After a mean observation period of 3.98 years, of the 332 restored teeth, 23 restorations showed failures (6.9%), 8 restorations (2.4%) showed major fractures, 11 restorations (3.3%) showed minor fractures, and 4 restorations (1.2%) failed due to secondary caries. The reported annual failure rate was approximately 2.2% per restoration. AP-X has a high filler, 70 vol%, compared to HMR, and does not have pre-polymerized clusters, which results in different mechanical properties. The stiffness (15.3 vs. 3.1 GPa), the flexural strength (190 MPa vs. 93 MPa) (Gee and Pallav, 1994; Mair, 1992; Muench et al., 2005; Venturini et al., 2023), and observed three-body wear (33 vs. 53 μm/200,000 rev) are important factors in the clinical behavior of these materials and may explain the difference between the results of Bartlett and Sundaram (2006) and Hamburger et al. (2011). In addition to these mechanical properties, the deviating fatigue wear behavior (2.8 vs. 7.2 mμ in two-body wear/200,000 rev) of HMR likely influenced the clinical results of HMR and the conclusions drawn by Bartlett and Sundaram. Their study suggests that resin composites are contraindicated for the treatment of severely worn posterior teeth when using HRM but not resin composites in general. To date, no published studies have examined the behavior of FURE in similar patient groups. However, based on the results of this study, the clinical performance of FURE is expected to be more likely to be similar to AP-X than to HMR. In contrast to AP-X, FURE, and HMR, CPB is an anterior composite intended for veneering teeth. Due to its lack of radiopacity, it is ideal for veneering posterior teeth, as this enables radiographic examination of approximal surfaces. In addition to this unique limited indication, the two- and three-body wear rates were low, and chip fractures were also rarely observed in patients with parafunctional habits. The wear rates of CME2 and HMR are relatively high; however, it is a universal resin composite suitable for anterior and posterior teeth. Both composites contain pre-polymerized clusters or agglomerates, which are porous in nature. As a result of these porosities and gaps, water penetration into the composite induces stress, leading to swelling, a reduction in the elastic modulus, and an increase in wear rates.

In addition to the wear of the restorative materials, the wear of the antagonist can also be a concern. Previously, we have shown that contact wear between various composites is important, especially when the filler in the composite is relatively hard and large (Osiewicz et al., 2022). In this study, the particles are generally soft (barium glass) (AP-X, CPB, and CME2) or have nano-sized particles (FURE). The roughness of the surfaces, based on the SEM and profilometric observations, of the two-body wear is lower than that of the surfaces of the three-body wear. This is in line with previous SEM and profilometric observations. As none of the investigated materials have large and hard particles, extensive wear of an antagonist is not expected.

*In vitro,* wear studies are valuable for understanding the principles of wear characteristics under controlled conditions, but they have several limitations, such as differences from oral conditions, the influence of saliva, temperature, and pH changes, and the inability to replicate long-term results, such as the wear of 10-year-old composites. In this study, we investigated only wear, roughness, and SEM analysis because our primary focus was on the effect of fatigue related to the stiffness of the materials. Hardness measurements of the composites were previously investigated as a function of filler load and composition.

As the stiffness of a resin composite influences its wear behavior, further research should focus on the optimal E-modulus of resin composites.

## Conclusion

The wear of the 1.5-mm specimens was significantly higher than that of the 3.0-mm specimens, showing for the first time that fatigue wear plays a role in the wear mechanism of dental resin-based composites. Therefore, the deformation of restorations under loading should be minimized. This can be achieved by using resin composites with high E-moduli, avoiding thin restorations, and preventing potentially flexible circumstances, such as the use of flowable liners under restorations.

## Data Availability

The raw data supporting the conclusions of this article will be made available by the authors, without undue reservation.
